# Comprehensive genomic profiling of the lung transcriptome in emphysema and idiopathic pulmonary fibrosis using RNA-Seq

**DOI:** 10.1186/1753-6561-6-S6-P21

**Published:** 2012-10-01

**Authors:** Rebecca L Kusko, John Brothers, Gang Liu, Lingqi Luo, Brenda Juan Guardela, John Tedrow, Yuriy Aleksyev, Ivana V Yang, Mick Correll, Mark Geraci, John Quackenbush, Frank Sciurba, Marc Lenburg, David A Schwartz, Jennifer Beane, Naftali Kaminski, Avrum Spira

**Affiliations:** 1 Genetics and Genomics Graduate Program, Boston University School of Medicine, Boston, MA, USA; 2 Bioinformatics Program, Boston University, Boston, MA, USA; 3 Division of Computational Biomedicine, Department of Medicine Boston University School of Medicine, Boston, MA, USA; 4 Simmons Center for Interstitial Lung Disease and Department of Medicine, University of Pittsburgh Medical Center, Pittsburgh, PA, USA; 5 Department of Pathology and Laboratory Medicine, Boston University School of Medicine, Boston, MA, USA; 6 Center for Genes, Environment and Health and Department of Medicine, National Jewish Health, Denver, CO, USA; 7 Dana-Farber Cancer Institute and Harvard School of Public Health, Boston, MA, USA; 8 Department of Medicine, University of Colorado School of Medicine, Aurora, CO, USA

## Background

As part of the Lung Genomics Research Consortium (LGRC), we sought to characterize transcriptomic alterations underlying the molecular pathogenesis of emphysema and idiopathic pulmonary fibrosis (IPF) using mRNA-Seq,and comparing to mRNAand microRNA microarray data obtained from the same samples.

## Methods

87 LGRC lung tissue samples were sequenced on the Illumina GAIIx, generating 75 nt paired-end reads and approximately 30-40 million reads per sample. Using gapped aligner Tophat, an average of 85% of reads aligned to hg19. Gene expression was quantified using Cufflinks and Ensembl59 known gene annotation (*n* = 24,249 genes). All lung tissue samples used in this study, as well as additional LGRC lung tissue samples, were run on Agilent V2 human whole genome arrays and Agilent V3 human miRNA microarrays.

## Results

Using a mixture model, expression of 6,359 genes was detected, 9,538 genes were not detected, and 8,397 genes were variably detected across all lung tissue samples. Using a subset of 58 samples from subjects with IPF (*n* = 19), emphysema (*n* = 19) or control samples (*n* = 20), differential gene expression was determined using a *t* test to compare each disease state with control. The expression levels of 1,770 genes differed between IPF and control, and 220 genes between emphysema and control (*P* < 0.001). Genes that go up in both emphysema and IPF relative to control were enriched for the p53/hypoxia pathway (KEGG, Biocarta) by GSEA. Gene expression estimates were highly correlated between the mRNA-Seq and array datasets across the same samples; however, we identified additional gene expression changes by RNA-Seq (either not significant by or not assayed by microarray) and validated these by quantitative PCR. Array-based gene expression estimates from additional lung tissue samples not sequenced, together with immunohistochemistry, confirmed the upregulation of the p53/hypoxia pathway in emphysema and IPF. Using reads that aligned across known splice junctions, we identified 5 emphysema-associated and 19 IPF-associated alternative splicing events. These events included the loss of exons and changes to the 3' UTR. Using qPCR and Nanostring, we validated two examples of differential splicing, one of which is shared by both chronic obstructive pulmonary disease (COPD) and IPF and involved with the p53 pathway. Finally, using miRconnX, miRNA microarray data and mRNA-Seq, data were integrated with a prior network of computationally predicted and experimentally validated miRNA-mRNA interactions. miRNAs that co-vary with differentially expressed p53/hypoxia genes in IPF and emphysema were identified and validated using *in vitro* miRNA perturbation studies.

## Conclusions

Our data indicate that the lung transcriptome is altered in emphysema and IPF, and suggest that these changes may include alterations in gene expression that are regulated by miRNA as well as disease-associated alterative splicing events that are shared between COPD and IPF. Specifically we observed a shared upregulation of the p53/hypoxia pathway and decreased expression of the miRNAs that may regulate this pathway. Our data also reveal disease-associated changes in known splice junctions that that may affect gene regulation or protein function. This unprecedented high-resolution view of the lung transcriptome associated with IPF and COPD may ultimately provide biomarkers of risk and response to therapy as well as potential therapeutic targets.

